# Are we measuring what matters? Aligning research outcomes with patient priorities in respiratory health

**DOI:** 10.36416/1806-3756/e20260172

**Published:** 2026-05-22

**Authors:** Juliana Carvalho Ferreira, Cecilia María Patino

**Affiliations:** 1. Methods in Epidemiologic, Clinical, and Operations Research-MECOR-program, American Thoracic Society/Asociación Latinoamericana del Tórax, Montevideo, Uruguay.; 2. Divisão de Pneumologia, Instituto do Coração, Hospital das Clínicas, Faculdade de Medicina, Universidade de São Paulo, São Paulo (SP) Brasil.; 3. Department of Preventive Medicine, Keck School of Medicine, University of Southern California, Los Angeles (CA) USA.

## PRACTICAL SCENARIO

While carrying out a prospective cohort study of mechanically ventilated ICU patients, investigators measured the occurrence of clusters of a particular type of patient-ventilator asynchrony (double triggering) and its association with endpoints typically reported in critical care research.^1^ They found that patients with these clusters (defined as ≥ 6 events per minute for at least 3 minutes) had longer durations of mechanical ventilation and ICU stays compared to those without it. A high cumulative duration of clusters (≥ 12 hours) was independently associated with worse clinical outcomes, including fewer ventilator-free days and reduced 28-day survival. The study endpoints are very objective and easy to measure, and while the study shows that physiological events such as double triggering are associated with traditional endpoints-including ventilator-free days-such measures do not capture how patients feel or function during and after ICU stay.

## WHAT DO PATIENTS VALUE?

There is no discussion about the value of surviving an ICU or a hospitalization stay for exacerbated COPD, and patients more often value subjective endpoints with an impact on lived experience than physiological endpoints, an intervention. Patients may be more interested in the impact of a new intervention that affects outcomes related to their symptom relief (dyspnea, fatigue), functional capacity, ability to perform daily activities, quality of life, and avoidance of exacerbations and hospitalizations. This mismatch between researchers’ priorities and patients’ expectations can impact on the true benefit of new interventions in real life.

## TRADITIONAL VS. PATIENT-CENTERED OUTCOMES

Common endpoints in respiratory research include FEV_1_, oxygenation, duration of mechanical ventilation, and apnea-hypopnea index. However, such endpoints do not always reflect meaningful patient benefit. These so-called surrogate endpoints^2^ ^,^ ^3^ often have a weak correlation with symptoms and quality of life, and small changes in such variables, although sometimes statistically significant, may lack clinical relevance (e.g., physiological improvement with bronchodilators in COPD with no perceived symptom benefit). In our practical scenario, it is possible that, for many patients, ventilator-free days are not as important as their ability to perform activities of daily living independently after ICU discharge.

Patient-centered outcomes are endpoints that reflect how patients feel, function, or live. Examples include subjective endpoints reported by patients themselves by means of quality of life questionnaires and validated tools used to assess symptom burden, such as the COPD Assessment Test (CAT) and the modified Medical Research Council (mMRC) dyspnea scale to quantify dyspnea. Although these measurements are sometimes considered less robust because they are subjective, context-dependent, and more variable than physiological endpoints, they measure outcomes that affect patients’ lives and are easy to understand; therefore, they have greater clinical relevance.

In order to design clinical studies that provide meaningful information about the impact of new interventions that matter for patients, the choice of study outcomes needs to be carefully considered. Researchers should engage patient representatives in all phases of a clinical trial, from study design to interpretation of results, and include patient-centered outcomes as endpoints. Clinical practice guidelines should also include patient-centered outcomes as critical outcomes to ensure that recommendations are based on outcomes that matter to patients. In addition, clinicians should be encouraged to interpret trial results beyond statistical significance, focusing on real-life impact and relying on such outcomes to inform patients about the benefits and risks of new interventions.

[Table t1]

**Table 1 t1:** Traditional vs. patient-centered outcomes in respiratory research.

Traditional outcome	Patient-centered outcome
FEV_1_	Dyspnea, symptoms (CAT)
DL_CO_	Exercise tolerance, fatigue (mMRC)
PaO_2_ / Oxygen saturation	Breathlessness scale
Radiologic progression (e.g., CT findings)	Quality of life
Lung compliance	Work of breathing, comfort
Eosinophil count	Symptom control, exacerbation frequency
Ventilator-free days	Functional recovery, independence, quality of life after ICU
ICU length of stay	Return to baseline life
Apnea-hypopnea index	Daily somnolence, quality of life

CAT: COPD Assessment Test; and mMRC: modified Medical Research Council Dyspnea Scale.

## KEY MESSAGES

- Measuring meaningful outcomes is essential for high-quality health care
- Overreliance on physiological endpoints shifts the focus away from patients toward surrogate measures, yielding results that have limited impact on patients’ lived experiences
- Clinical research should integrate patient perspective into research design and interpretation
- Necessity to align research outcomes with patient priorities to improve real-world impact
